# An Integrated Care Approach to Improve Well-Being in Breast Cancer Patients

**DOI:** 10.1007/s11912-024-01500-1

**Published:** 2024-02-24

**Authors:** Alessandra Fabi, Alessandro Rossi, Edoardo Mocini, Ludovica Cardinali, Valerio Bonavolontà, Cristina Cenci, Stefano Magno, Vittoria Barberi, Antimo Moretti, Zein Mersini Besharat, Giovanni Iolascon, Carlo Baldari, Elisabetta Ferretti, Andrea Botticelli, Ida Paris, Giovanni Scambia, Silvia Migliaccio

**Affiliations:** 1 Precision Medicine Unit in Senology, Fondazione Policlinico Universitario A. Gemelli IRCCS, Largo Agostino Gemelli 8, 00168 Rome, Italy; 2 Department of Movement, Human and Health Sciences, University Foro Italico of Rome, Rome, Italy; 3 Department of Experimental Medicine, University Sapienza of Rome, Rome, Italy; 4 Bone Metabolic Diseases Unit, Department of Experimental and Clinical Biomedical Sciences, University of Florence, Florence, Italy; 5 Department of Biotechnological and Applied Clinical Science, University of L’Aquila, L’Aquila, Italy; 6 DNM-Digital Narrative Medicine, Rome, Italy; 7 Center for Integrative Oncology, Fondazione Policlinico Universitario A. Gemelli IRCCS, Rome, Italy; 8 Medical Oncology 1, Regina Elena National Cancer Institute IRCCS, Rome, Italy; 9 Multidisciplinary Department of Medical and Surgical Specialties and Dentistry, University of Campania “Luigi Vanvitelli”, Caserta, Italy; 10 Department of Theoretical and Applied Sciences, eCampus University, Rome, Italy; 11 Department of Radiological, Oncological and Pathological Sciences, Policlinico Umberto I, Sapienza University of Rome, Rome, Italy; 12 Department of Woman and Child Health and Public Health, Fondazione Policlinico Universitario A. Gemelli IRCCS, Rome, Italy

**Keywords:** Breast cancer, Multidisciplinary approach, Tailored oncology, Cancer prevention, Oncology and metabolism, Personalized medicine, Integrative oncology

## Abstract

**Background:**

Breast cancer (BC) treatment has recently been revolutionized by the introduction of newer targeted agents, that helped tailoring therapies around the single patient. Along with increased survival rates, a careful evaluation of diet, lifestyle habits, physical activity, emotional and psychological experiences linked to the treatment journey, is now mandatory. However, a true proposal for an omnicomprehensive and “integrative” approach is still lacking in literature.

**Methods:**

A scientific board of internationally recognized specialists throughout different disciplines designed a shared proposal of holistic approach for BC patients.

**Results:**

A narrative review, containing information on BC treatment, endocrinological and diet aspects, physical activity, rehabilitation, integrative medicine, and digital narrative medicine, was developed.

**Conclusions:**

In the context of a patient-centered care, BC treatment cannot be separated from a patient’s long-term follow-up and care, and an organized interdisciplinary collaboration is the future in this disease’s cure, to make sure that our patients will live longer and better.

**Trial Registration:**

*NCT05893368*: New Model for Integrating Person-based Care (PbC) in the Treatment of Advanced HER2-negative Breast Cancer (PERGIQUAL). Registration date: 29th May 2023.

## Introduction

Breast cancer (BC) represents the first most prevalent neoplasm worldwide and one of the leading cause of death in the female gender [1]. The proportion of intrinsic (e.g. random genomic alterations arising during DNA replication) and extrinsic (e.g. mutations that arise owing to environmental exposure) factors that determine its development is still a matter of debate. However, analysis of mutational fingerprints suggested that up to 65% of BCs present extrinsic mutational signatures, indicating considerable contribution of environmental exposures/lifestyle habits in the development of this disease [2].

Obesity is a risk factor for cancer development and associated with cancer-related mortality in different tumors, including BC [3]. Although the epidemiological association among obesity and lifestyle habits (as alcohol, smoking, and sedentary behavior) with many forms of cancer is clear enough, the biological relationship between carcinogenesis and these risk factors remains poorly understood [4].

In addition, several environmental pollutants (Cadmium, BBA, etc.), which are released after industrial processes and absorbed into certain plants and water, have recently been considered as additional risk factors for several different hormonal responsive cancers [5, 6]. Indeed, our group has demonstrated as Cadmium might alter breast cancer responsiveness in vitro, (Bimonte et al. Endocrine, in revision) leading to even more complex pathogenetic mechanisms in the hormonal carcinogenesis and therapeutical approach.

The treatment landscape of BC is constantly evolving, with pharmacological interventions which have reached impressive results both in early and advanced settings [7•]. According to molecular features (e.g. estrogen receptor, HER2 status, PDL1 expression, and BRCA mutation), defined sequences and/or combination of target therapy, anti-hormonal therapy, immunotherapy, and/or chemotherapy can be offered to patients [8–19]. The development of new treatments unavoidably led to different toxicity profiles, which means that there is a true need of an organized network of professional healthcare figures involved in the patients’ treatment path. Moreover, improved survival outcomes recently translated into particular attention for quality of life (QoL) [20, 21].

Evidences of an integrated and omnicomprehensive approach are still lacking in literature, since available data are only focused on prevention or provide only a few useful hints for breast cancer patients [22, 23].

Therefore, a holistic approach might be a quite powerful and effective tool, beside pharmacological intervention, not only to help tailoring the treatment for each patient but also to improve QoL. Herein, we provide a narrative review on an “integrative medicine” model, based on an interdisciplinary approach that might help achieving improvement in survival and QoL outcomes for women affected by BC.

## Methods

This narrative review is the result of an intense collaboration between specialists across different disciplines, which shared their scientific and clinical experience to develop information and hints on all aspects of a BC patient treatment journey. Several detailed paragraphs have been developed by specific experts to explore the latest available data for each discipline and suggest how to properly manage a BC patient.

## Nutrition and Metabolism in Breast Cancer

Patients undergoing BC treatments, such as Chemotherapy (CHT), Antibody–Drug Conjugates (ADCs), and Radiation Therapy (RT), face nutritional and metabolic challenges. CHT often carries nutrition related symptoms, such as nausea and vomiting [24]. Antibody–Drug Conjugates (ADCs) and target therapies may also cause similar effects and altered taste or smell perception as well as oral mucositis [25]. Damage to taste receptor cells, along with zinc and other heavy metal chelation, contributes to loss of taste. Radiation therapy (RT) frequently damages salivary glands, altering the composition and quantity of saliva, favoring xerostomia. All these side effects, among others, may lead to malnutrition [26] that negatively affects QoL and survival, contributing to other conditions such as cardiovascular diseases and treatment-related acute events [27]. CHT can also disrupt glucose metabolism [27, 28], leading in some patients to insulin resistance, which contributes to increased body weight, generally characterized by excessive fat mass with loss of muscle mass, a condition known as sarcopenic obesity (SO) [27]. This condition is further exacerbated by reduced PA due to treatment-induced side effects like asthenia and fatigue [26]. Moreover, the use of corticosteroids and cytotoxic agents affects the maintenance of proper weight and body composition as well, increasing the risk of SO. Also estrogen deprivation due to adjuvant endocrine treatments for Hormone Receptor Positive (HR +) BC and/or CHT can affect body composition, with alteration in skeletal, adipose, and muscle tissues, leading to sarcopenia, obesity and osteoporosis [29]. Obesity at diagnosis and body weight gain after treatment are frequent and have been linked to higher BC recurrence and mortality rates and to lower QoL [30].

On the other hand, osteoporosis, a known side effect of adjuvant hormonal therapy for breast cancer, increases the risk of fragility fractures, impacting long-term morbidity and QoL. In addition to that, the decline in muscle strength and quality due to sarcopenia, possibly caused by the mechanisms previously described, can also affect the risk of falls and consequent fragility fractures necessitating a comprehensive treatment approach, including nutritional interventions [31]. This is particularly important for HR + BC patients, where managing nutritional intake is vital due to the significant concern of hormonal imbalances that might contribute to the previously described mechanisms. Interestingly, several studies have found a link between BC, diet and life-style. The latest studies have focused on the identification of risk factors that could contribute to unfavorable outcome of BC, thus in 2018 the World Cancer Research Fund has produced a report showing the latest and significant evidence to improve the overall survival through lifestyle recommendations [32]. Indeed, lifestyle improvement has a significant impact on BC management after initial diagnosis, both in term of therapeutical efficacy and QoL [33]. Despite the overall nutritional healthy choices, the maintenance of a normal body weight is mandatory, since it is well documented that obesity is linked to worst outcomes [34, 35]. For these reasons, particular attention must be paid to meeting the protein and caloric needs as part of managing weight and body composition, as mentioned before, a crucial aspect of BC treatment. Achieving and maintaining an optimal body weight in BC patients is a priority for the whole care team both by correct healthy diet but also by appropriate PA. The principles of the MeDi, with its balanced approach to macro and micronutrient intake, are highly recommended for their beneficial effects on glucose and insulin levels, as well as overall metabolic health [36, 37•]. In contrast the Western Diet, with high consumption of fats, activating pathways involving insulin and IGF-1 which, when combined with sedentary life, could worsen outcomes of BC patients [38]. The role of MeDi is due to the food of which this nutritional pattern is composed. MeDi includes a daily consumption of vegetables and fruits that allows an optimal intake of polyphenols and fiber [39, 40]. Interestingly, a recent review indicated that a higher fruit, vegetable, and fiber intake and a moderate soy/isoflavone consumption were associated with beneficial outcomes in BC survivors [40]. A small consumption of dairy product is contemplated in the MeDi pattern, but dairy products are often avoided by women due to the fear of a potential worsening of the disease. Interestingly, some studies have indicated that the consumption of a modest amount may reduce the risk of BC; nonetheless, some dairy products contain a high percentage of saturated fatty acids with pro-carcinogenic effects. No recommendations can also be made for the soy products [41, 42]. Soy foods contain isoflavones that have weak estrogen-like activity, but it has anti-carcinogenic and antioxidant properties Thus, at the present time, it is recommended to follow MeDi indications [41, 42]. Carbohydrates should be present but whole grain bread and whole meal products should be chosen to decrease the glycemic index [43•]. MeDi limits the intake of meat and animal food. Even though few studies have evaluated in BC survivors whether red and processed meat increase or worsen cancer, it is advised to follow MeDi indication and limit this type of food.

As mentioned, it is not possible to find an association between specific nutrients and BC, except for alcohol. Alcohol is the only nutrient with demonstrated negative effects on BC, confirmed in every study [44, 45]. This risk is not associated to the type of alcohol consumed and the patients’ age, since ethanol promotes tumor growth and metastasis formation, and apparently increases the effects of estrogens on breast tissue through several pathways [46].

It is also important to note that BC patients may also autonomously modify their diets, following anecdotal advices and common beliefs or online and social media sources [47], to try cope with physical and psychological consequences of their condition. Orthorexia nervosa (ON) is a condition where patients become overly preoccupied with eating “healthy” foods, often leading to dangerous exclusions potentially resulting in malnutrition [48]. ON risk has been found higher among BC patients compared to healthy controls [49]. In this context, clinical nutrition specialist plays a crucial role in addressing nutritional issues and ensuring comprehensive care, guiding patients in becoming aware of their specific needs, offering tailored advice and support.

To sum up, nutritional assessment and therapy is essential for addressing the adverse effects of BC treatments and enhancing their efficacy, as well as improving patient outcomes. The comprehensive management of BC necessitates an integrative approach that combines structured nutritional strategies and professional counseling for lifestyle modifications. This holistic approach is crucial for enhancing the therapeutic efficacy and improving the overall outcomes for patients, particularly in the context of HR + BC. Nutritional care extends beyond merely defining a proper diet. It involves assisting patients in finding personalized strategies to optimize their nutrition within their daily lives and monitoring their nutritional status to identify early signs of malnutrition or related conditions such as sarcopenia, obesity, or symptoms of eating disorders. Promptly addressing these conditions through early interventions is vital for effectively treatment or management, highlighting the importance of nutritional care in BC treatment.

## Integrative Treatments in Early and Advanced Disease

QoL during and beyond cancer treatments is a key aspect in the large population of BC survivors, either for the physical and psychological burden of the disease experience and the numerous side effects and symptoms [50, 51], even with late onsets, potentially affecting patients’ adherence to anticancer treatments [52–54].

Most of the early and late side effects are not completely assessed and properly met among BC patients/survivors, who may turn to “alternative medicine” practitioners outside the comprehensive cancer centers, with potential risks for drug interactions, unproven treatments and eventually threats to their health [55].

Worldwide, an estimated 33–47% of oncological patients use complementary, alternative, or integrative medicine during their therapeutic process, while more than 80% of BC survivors reported using integrative therapies during and beyond cancer treatments [56–58].

The Integrative oncology, according to the definition approved by a panel of multi-disciplinary experts, is a “patient-centered, evidence-informed field of cancer care that utilizes mind and body practices, natural products, and/or lifestyle modifications from different traditions alongside conventional cancer treatments”. Accordingly, it combines lifestyle counselling, body-mind activities, and complementary therapies with anticancer standard care, to improve management of symptoms, adherence to the treatment protocols, improve QoL, clinical outcomes across the cancer continuum, and empower people preventing cancer and become active participants before, during, and beyond oncological treatment” [59].

Despite the widespread use of integrative treatments and the available evidence about their safety and efficacy [60], facilities and services providing those treatments inside the BC centers are lacking [61], at least in Europe, while many of the National Cancer Institute designated comprehensive cancer centers already offer some integrative therapies in the United States.

A holistic model should include a comprehensive assessment for each patient after cancer diagnosis, either waiting for surgery or neoadjuvant chemotherapy, with a preliminary psycho-oncological distress evaluation and a brief interview about lifestyles, to identify psychological and/or physical needs that may affect patient’s compliance to treatments.

To be accepted by the scientific community as a reliable answer to the multiple unmet needs of BC patients, the integrative model must be safe, rational and evidence based. Moreover, the specific interventions should be prescribed by integrative oncology practitioners rather than on demand by the patient, who might otherwise hold incorrect information from unreliable sources often failing to distinguish between “integrative/complementary” and “alternative” cures.

According to the SIO/ASCO clinical guidelines, complementary approaches for BC patients should include acupuncture, massage therapy, mindfulness-based protocols, and music therapy, tailored to the clinical needs summarized in the following lines.

Acupuncture represents a reliable, cost-effective, and safe procedure for symptom management, if performed properly by a specialized practitioner. It can be recommended for chronic pain, fatigue, nausea/vomiting and hot flashes, as an option to avoid or reduce pharmacological treatments, or for other conditions (fatigue, hot flashes, and CHT-induced peripheral neuropathy) for which conventional treatments are ineffective, not available or burdened by remarkable side effects.

Even though acupuncture alone is debated for cancer pain, it could be recommended in frail patients with comorbidities and when side effects of drugs, such as opioids, are relevant. According to the Society for Integrative Oncology-ASCO guidelines about integrative medicine for pain management in oncology, acupuncture should be recommended for aromatase inhibitor-related joint pain in breast cancer patients and survivors [62]. CHT-induced peripheral neuropathy (CIPN) is a challenging symptom, and the role of acupuncture is still uncertain [63]; nevertheless, some randomized studies showed promising results [64–66], and acupuncture might be considered in selected patients to treat CIPN symptoms according to the ESMO guidelines [67]. Among the massage therapy procedures, reflexology is a safe and well tolerated technique which can be used to alleviate pain, anxiety [68] and sleep disorders that are poorly controlled by pharmacological treatments [69].

Mindfulness-based stress reduction (MBSR) techniques have been shown to reduce distress and improve primary insomnia and psychological well-being in patients with cancer [70, 71]. In randomized trials based on MBSR programs women reported decreased fatigue, depression, anxiety, fear of recurrence and health related QoL [72, 73]. Mindfulness and hypnosis may improve QoL, fatigue and anxiety even in advanced breast cancer patients [74].

Music therapy has been shown beneficial on depression, pain, and cancer-related fatigue during and after treatments, either through active (e.g., singing, using percussive instruments, and playing with sounds) and passive (e.g., listening) techniques [75, 76]. Music therapy may also be recommended for reducing anxiety in the perioperative setting, among patients undergoing mammographic screening, during radiation, and CHT sessions [77]. In the advanced stages of the disease, integrative approaches could represent an additional tool for the oncology health-care providers, who struggle with managing pain, complex side effects or toxicities, and drug interactions using conventional treatments. According to the 5th ESO-ESMO international consensus guidelines, complementary therapies have the potential to reduce disease symptom burden and/or side-effects of anticancer therapies and therefore improve the QoL of advanced breast cancer patients [74].

**Fig. 1:**
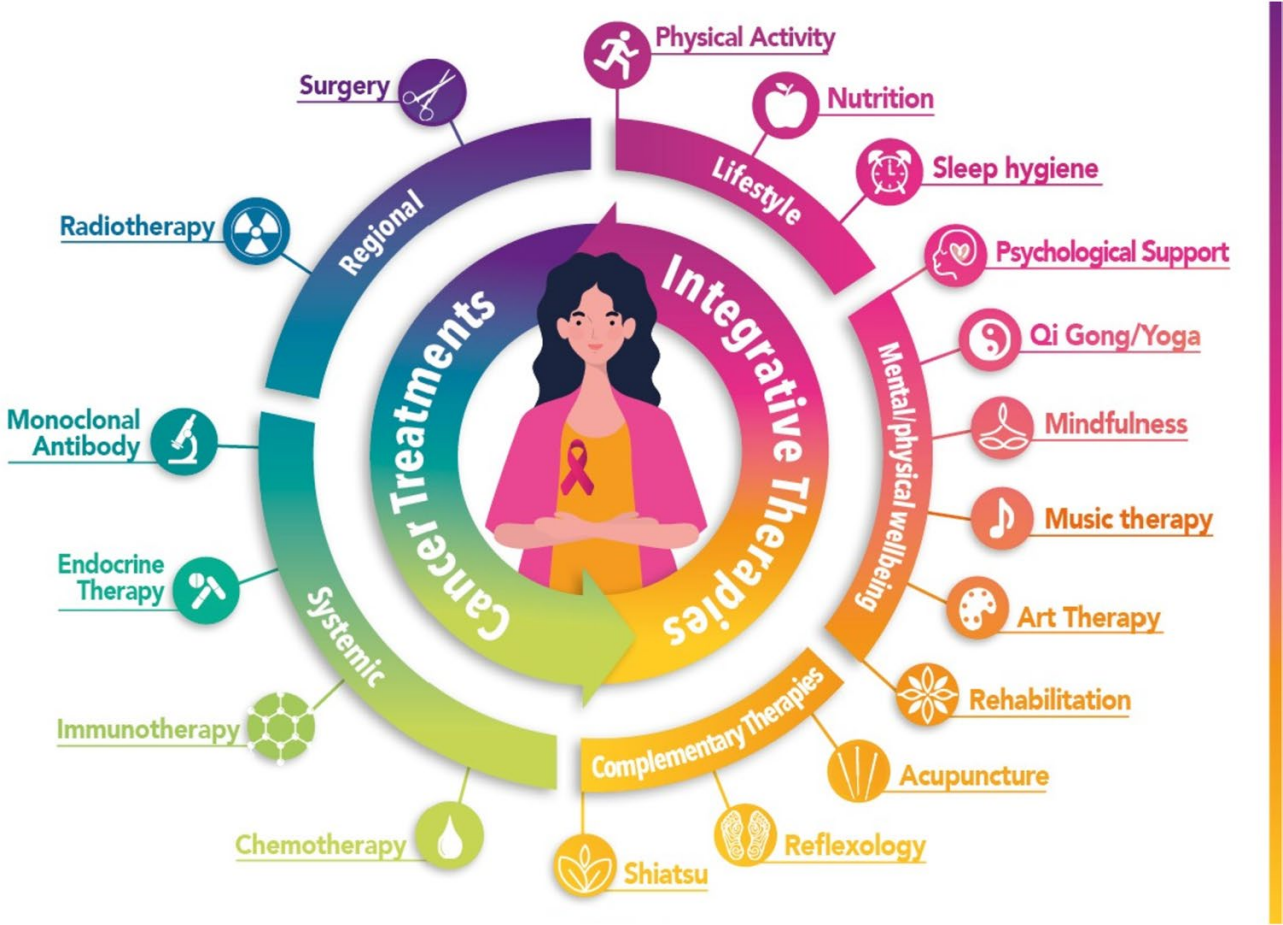
Integrative oncological approach for early and advanced breast cancer

Figure 1 shows our proposal for a holistic model of cancer care continuum, in which integrative and supportive approaches are combined with anticancer treatments.

## The Role of Rehabilitation, Physical Activity, and Exercise

Rehabilitation and physical activity might be part of a unique therapeutical pattern, even though they have quite different aims. In fact, while rehabilitation might play a pivotal role soon after surgery, physical activity may play an important role for the maintenance of optimal body composition and quality of life during or after pharmacological treatments.

Indeed, it is estimated that 60–90% of cancer patients report some degree of mobility and independence problems; therefore, cancer rehabilitation, performed in its various forms and settings, may help enhancing physical, psychological functioning, and social interactions, providing a better QoL [78, 79].

During the disease, due to BC itself or to medical/surgical treatments, patients experience a series of impairments and consequent disabilities that require intervention for pain control, mobility improvement, lymphedema, and restoration of independence in activities of daily living (ADL) [80].

Rehabilitation is a multidisciplinary process that involves different healthcare professionals, such as a physiatrist, physical therapist, and occupational therapist [81, 82]. The care plan begins with a multidimensional assessment, which identifies the problems in terms of impairments and disabilities, proceeding to formulating an individual rehabilitation plan (IRP) based on a biopsychosocial approach, according to the WHO-International Classification of Functioning, Disability, and Health (ICF) framework [83]. For practical purposes, it has proposed ICF Core Sets, namely, the salient ICF categories/codes of functioning to be rated in all patients with BC to guide multidisciplinary assessments of impairments of body structure (e.g., increased volume of upper limb treated with lymphadenectomy) and/or body functions (e.g., pain of upper limb treated with lymphadenectomy), limitations in ADL and restrictions in social participation (e.g., lifting and carrying objects), including the contextual factors involved in the management of this population (e.g., caregiver burden) [83]

Table 1 depicts an example of the operative procedure of the rehabilitation approach for a BC patient. In fact, it is possible using the ICF, to individually approach rehabilitation of the patients, depending on the individual health and health-related domains regarding individually functioning and disability.

**Table 1:** Example of rehabilitation procedures for BC patients

ICF code	ICF category	Outcome measure	Rehabilitation intervention	Expected outcome
b28014	Sensation of pain in upper limb	Numerical Rating Scale (NRS)	Analgesics and transcutaneous electrical nerve stimulation (TENS)	Pain relief
b710	Mobility of joint functions	Active and Passive Range of Motion (ROM)	Flexibility exercises (e.g., ROM exercises and joint mobilization)	Mobility and motility improvements
b730	Muscle power functions	Manual muscle testing (MMT)	Resistance/strengthening exercise	Improvement of muscle strength
s730	Structure of upper extremity	Tape measure circumference	Complete decongestive therapy (CDT)	Reduction of upper limb circumference
d430	Lifting and carrying objects	Disabilities of the Arm, Shoulder, and Hand (DASH) Questionnaire	Flexibility exercises, Resistance/strengthening exercise, Occupational Therapy (OT)	Improvement in performing daily activities involving the upper limb (e.g., lifting a cup or carrying a box from one room to another)
d445	Hand and arm use	DASH Questionnaire, Barthel Index (BI)	Flexibility exercises, resistance/strengthening exercise, OT	Improvement in performing daily activities involving the upper limb (e.g., turning door handles or catching an object)
d640	Doing housework	Eastern Cooperative Oncology Group (ECOG) Performance Status, BI	Flexibility exercises, resistance/strengthening exercise, OT	Improvement in managing household (e.g., washing clothes, storing food and disposing of garbage, tidying rooms, closets and drawers; collecting, washing, drying, folding and ironing clothes)
e310	Immediate family	Caregiver Strain Index (CSI)	Educational program to improve skills in meal support, nursing care, welfare care, or symptom management	Reduction of the level of stress of family caregiver (e.g., parents and partner)
e580	Health services, systems, and policies	Rehabilitation Complexity Scale (RCS)	Care coordination	Facilitation of the access to multidisciplinary health services to better manage health-related issues

Rehabilitation interventions are indicated in several moments of the care pathway of BC patients. For instance, primary prevention refers to measures designed to avoid the development of risk factors or disease occurrence, with PA that is highly recommended for these purposes [84, 85].

A specific role is recognized in rehabilitation for women undergoing surgery, namely, “pre-habilitation” [86]. This approach should be implemented as soon as the surgical indication has been established to improve not only the short-medium term functional items but also the surgical outcomes in terms of clinical, functional, psychological, and post-operative complications [87, 88]. Pre-habilitation has also a key role in women who will undergo radio- and/or CHT, which is known to lead to significant musculoskeletal impairments, including widespread pain (e.g., joint pain and myalgia), muscle wasting or cachexia, bone fragility, and cardiorespiratory deconditioning [89, 90].

Rehabilitation programs are both useful in hospitalized and outpatient settings, as reported by several studies with significant improvements in functioning, cognitive status, QoL, and social participation [91].

The COVID-19 pandemic has led to the rapid development of telerehabilitation for BC patients, which in a recent systematic review provided not otherwise possible access to care and reduced cancer-related disability [92].

After the conclusion of the rehabilitation phase, the patient can start a physical activity program, along with a dedicated nutrition and exercise plan. Indeed, PA is well recognized as a safe, effective, and feasible intervention strategy that can reduce the number and severity of BC treatment-related side effects and symptoms (e.g. pain, fatigue, sleep disturbances, and cognitive impairment, neurological disorders), with a clear positive impact on survival [93, 94]. It was demonstrated that patients meeting or exceeding the minimum PA guidelines of ≥ 150 min of moderate to vigorous PA (MVPA) per week before and at 1 and 2 years after BC diagnosis experienced statistically significant reductions in hazards of recurrence and mortality [93]. Moreover, PA has also been reported to reduce the likelihood of metastatic spreading [95] and is associated with numerous other health benefits among BC survivors, including weight loss or maintenance, reduced depression and anxiety, management of post-treatment symptoms, mitigation of cancer-related fatigue, improved social support, and QoL [94]. The potential biological mechanisms with protective effects on BC include a reduction of sex and metabolic hormones, adipokines and oxidative stress, improvement of the immune function [95, 96]. Most health benefits induced by PA are associated with body mass index (BMI) and weight loss, indicating that the level of adiposity and the percentage of fat mass are critical indicators in determining the effect of exercise [97] Moreover, postmenopausal women treated with aromatase inhibitors (AIs) might experience severe arthralgias, osteopenia and osteoporosis, depression, hypertension, and sexual dysfunction [98], reported to be grade 3 or higher in up to one third of BC patients. This affects QoL [99] and might be positively modulated by a correct PA approach. Despite this knowledge, the level of PA is low among BC patients, with more than one-third of this population that prefers spending prolonged time sitting or participating in little or no leisure-time PA (LTPA) [100, 101]. Therefore, it is essential to introduce a qualified kinesiologist as part of the clinical team who can help start and maintain a safe exercise program by operating on behavior change strategies and administering an adapted PA (APA) plan [102]. Exercise prescription based on Frequency, Intensity, Time, Type (FITT) parameters should be preceded by an assessment of all components of health-related fitness (i.e., cardiorespiratory fitness, muscular strength and endurance, body composition, and flexibility) to create a personalized exercise program considering the individual needs of patients based on general recommendations [103].

The most recent PA guidelines for BC survivors emphasize the importance to “avoid inactivity” and be as physically active as possible, and recommend performing moderate-intensity aerobic training at least three times per week, for at least 30 min, two or more days a week of resistance training using two sets of 8 to 15 repetitions at least 60% of one repetition maximum (1RM), and daily stretching of major muscle groups, with specific exercise modifications based on health and cancer status and treatment-related side effects [103].

Despite most of the available literature has proposed effective exercise programs dealing mostly with aerobic exercise and/or resistance training using hand-held or machine weights or therabands, a growing body of studies has investigated the effects of innovative practices for BC patients including Pilates, Yoga, water-based exercise, Tai Chi, dragon boating, recreational sports, wall/rock climbing, and triathlon [103, 104], suggesting a potential additive positive effect on several issues.

A recent systematic review on the role of yoga in symptoms management for cancer survivors reported improved fatigue and QoL, whereas further research is needed to confirm the effects on improving sleep, depressive symptoms, anxiety/distress, and cancer-related cognitive change [105].

Similarly, mind–body approaches as Pilates and Tai Chi showed significant effects in females with BC in terms of functional capacity, fatigue, flexibility, and QoL improvement compared to a control group [106]. Even body composition, and QoL, as well as muscular, memory, and cognitive functions were improved [107, 108]. As suggested by Costa and colleagues [109], in the management of BC patients, it is crucial to verify the impact of a supervised group exercise intervention to improve aspects related to health, physical functioning, and QoL.

Rogers et al. [110] reported that 3-month PA intervention benefits on health persisted 9 months after the end of the intervention. Adherence and long-term benefits to both PA unsupervised and supervised physical exercise (PE) programs may represent a major issue for cancer survivors.

An intervention that comprises a supervised in-person PE program and active lifestyle recommendations is advisable in BC patients as it appears to be more effective than unsupervised or home-based programs (i.e., more attention, motivation, and reinforcement) [103, 109].

However, some factors such as on-site appointments, travel, time constraints, and lack of trained professionals can increase barriers to accessing physical activity for patients [111]. For these reasons, online PA interventions and wearables or smartphone applications may represent an additional support in the promotion of PA and PE in BC patients [111, 112].

Even though it is difficult to provide a general and definitive exercise prescription for BC patients and survivors, it is reasonable to propose a supervised multi-component PE intervention preferably in person (in condition of poor accessibility, an online supervised intervention may be a valid alternative) that is focused not only on aerobic exercise or a combination between aerobic and resistance exercise, but rather on improving all the components of physical fitness as well as on meditation, posture, core strength, and breathing techniques.

## e-PROMs and Digital Narrative Medicine: A Novel Person-Based Framework for Integrative Oncology

A comprehensive assessment of patient needs, habits, resources, difficulties, and expectations is key to foster Integrative Oncology.

Integrating therapies with a dietary plan, exercise programs, and all the different non-pharmacological options requires appropriate methodologies to detect the unique and unrepeatable subjectivity of everyone, associated not only with the psychological profile but also with the social and environmental context of reference.

The emerging paradigm of person-centered medicine has led to a change in the approach and management of health needs, introducing new models and tools for patient engagement in the care process. An important change is represented using the Patient Reported Outcome Measures (PROMs-e-PROMs), related to health status reported directly by the patient, without the mediation of the clinical interview that often leads to underestimating effects [113].

A randomized study conducted in patients undergoing CHT showed clinical benefits, including improved tumor survival, when symptoms reported systematically by the patient using a web-based monitoring system during treatment are taken into consideration in addition to standard reference parameters [114]. A review by ESMO has shown the benefits and provided indications for optimal use [115•].

However, PROMs and e-PROMS remain anchored to a purely clinical model. They do not enter the emotional and social territory of the specific subject that influences quality of life. PROMs gather information and not experiences; they identify mostly problems and not the bio-psychosocial resources of each individual [116].

For Integrative Oncology, the ICF serves as a reference framework not only for the rehabilitation but also for the development of and appropriate listening model. For each individual, the ICF distinguishes between capacity and performance. Capacity is the functioning of a patient assessed with clinical instruments, while performance is the lived experience of the person related to the global environment. Performance can be better or worse than capacity, depending on the personal, family, social, and environmental resources available. Detecting the specific mix of these resources is fundamental.

For this objective, personalized listening can be strengthened by the introduction of narrative medicine [117, 118] which enrich the point of view expressed in a standardized manner, favoring a *person-centered* approach rather than a *disease-centered* one.

The development of digital technologies represents an opportunity for the diffusion of narrative medicine in clinical practice, facilitating the listening of subjective needs and demands.

In 2016, the first digital platform designed for constructing narrative medicine pathways was launched in Italy. The platform's functionalities leverage the potential of narrative medicine for doctor-patient communication while preserving the confidentiality of health data. The IRCCS National Cancer Institute “Regina Elena” in Rome was the first to conduct pilot studies in the field of oncology, integrating digital narrative diaries into clinical practice for surgical and chemotherapy/radiotherapy-treated patients.

The results in terms of feasibility and usefulness have been very positive. The strength, as perceived by both clinicians and patients, lies in the ability to share relevant aspects of the patient's experiences and needs that would otherwise go unnoticed [119•, 120].

A new framework called PbC (Person-based Care) has been proposed to integrate e-PROMS and digital narrative medicine in the ongoing Pergiqual study (NCT 05893368) at the Fondazione Policlinico Gemelli in Rome. The approach involves integrating digital narrative listening and quality of life questionnaires (EORTC QLQ C-30 and B-23) to assess the impact of the disease and treatments, with the aim of personalizing the care pathway. The model includes an initial phase of narrative listening using a prompts-guided digital diary, followed by a baseline evaluation using e-PROMs of QoL before the start of therapy. This is followed by ongoing narrative monitoring throughout the course of therapy and follow-up, integrated with repeated assessments of quality of life using e-PROMs over time.

The results of the Pergiqual study will enable the multidisciplinary group to develop a dedicated digital environment that utilizes e-PROMs and narrative medicine in a cohesive patient journey.

## Conclusions

Since novel treatment strategies led to impressive survival outcomes for BC patients, QoL in now another key mandatory endpoint in clinical practice.

The chronicization of the disease, increasingly evident for many biological BC subtypes, requires now a multidisciplinary approach to address all the potential sequelae of the disease or therapies side effects, which could affect long-term QoL.

Approaching the patient with a holistic view is a major challenge today. The integrative therapies consider the patient as a whole, addressing not only the physical aspects but also emotional well-being.

This holistic approach can contribute to better QoL, and therefore survival, outcomes.

The “electronic health” (eHealth) technologies are now aiding clinicians to overcome physical barriers and distances, but more work is needed to ascertain and optimize effectiveness, measure long-term effects.

Moreover, in recent years, liquid biopsy and circulating biomarkers helped address medical needs by providing a non-invasive tool for patient in course of treatments and during follow-up. Although their role in BC prognosis has now been deeply evaluated [121], it is still not clear wheter this new non-invasive approach could be useful to address molecular benefits of holistic approaches on QoL and how can we integrate these techniques in machine-learning programmes [122].

Our manuscript focused on the need of an integrated management, from oncologic intervention to nutrition, physical activity, and rehabilitation. Interesting new models and tools for patient engagement in the care process, such as digital narrative medicine, can capture the patient’s experiences and its emotional needs. Continuous evaluation of new approaches will further help improving the management of this tumor but also detect early discomfort and maintain well-being of all our patients.

## Data Availability

As a review article, all data and materials are directly available from the authors upon specific request.
